
JOURNAL INFORMATION
==============================
NLM Title Abbreviation: ScientificWorldJournal
Journal ID: ScientificWorldJournal
NLM Journal ID: 101131163
Journal ID: TSWJ

The Scientific World Journal

ISSN: 2356-6140
EISSN: 1537-744X
Publisher: Wiley

ARTICLE INFORMATION
==============================
PMCID: PMC5848657
PMID: 18836663
DOI: 10.1100/tsw.2008.134
Article ID: 315439
Article version: 1
Subjects: Commentary

Nephrotoxicity of Mala Fide Melamine: Modern Era Milk Scandal

Lattupalli Rakesh *
Kolluru Anuradha
Yee Jerry
Division of Nephrology, Henry Ford Hospital, Detroit, MI, USA
*Rakesh Lattupalli: rlattup1@hfhs.orgAcademic Editor: Graham V. Lees

Publication date: 2008
Electronic publication date: 2008 Oct 1
Volume: 8
First page: 949
Last page: 950
Received 2008 Sep 30; Accepted 2008 Sep 30
Copyright: Copyright © 2008 Rakesh Lattupalli et al.
Copyright year: 2008
License: This is an open access article distributed under the Creative Commons Attribution License, which permits unrestricted use, distribution, and reproduction in any medium, provided the original work is properly cited.
License URL: https://creativecommons.org/licenses/by/3.0/

==============================
Commentary
*TheScientificWorldJOURNAL* (2008) 8, 949–950
ISSN 1537-744X; DOI 10.1100/tsw.2008.134

TheScientificWorldJOURNAL
www.thescientificworld.com

### Nephrotoxicity of Mala Fide Melamine: Modern Era Milk Scandal

Rakesh Lattupalli*, Anuradha Kolluru, and Jerry Yee

**E-mail**: RLATTUP1@hfhs.org

*Received September 30, 2008; Accepted September 30, 2008; Published October 1, 2008*

---

In 2007, a major pet food recall occurred because of an outbreak of kidney failure in cats and dogs that was linked to melamine in pet food[1]. The kidney failure was attributed to stone disease[2]. Notably, similar outbreaks in 2004, with identical findings, implicated a similar causation[3], and pork and poultry from animals fed with melamine-contaminated feed had also been suspected. However, based upon the limited information available at that time, melamine was deemed a very low human risk by the FDA and USDA[4]. Thus, without potential for human toxicity, melamine quickly exited the spotlight.

Melamine is used industrially as a plastic resin and is known for its fire-retardant properties. Its high nitrogen content fosters its illicit use as dietary nonprotein nitrogen. Melamine, added to food products, inflates their protein levels on standardized tests that quantify nitrogen content as a surrogate for protein.

Now, renewed interest in melamine comes from reports of acute renal failure, resulting in four infant deaths and thousands of hospitalizations in China from melamine in milk and infant formulas. These catastrophes have led to widespread public outcry and prompted product recalls.

The exact pathophysiology between melamine and kidney disease is unclear. Kidney biopsies from some animals with pet food–associated kidney failure revealed crystal involvement of distal tubules, fibrotic changes, and inflammation[3]. The presence of a cofactor may be required to induce kidney failure, and cyanuric acid, obtained from the same raw material where melamine is procured, has been proposed as one such factor[5]. However, the presence of cyanuric acid in infant formulas is unknown. Other unknowns remain regarding melamine-induced kidney injury: Is a certain subset of population like infants at an increased risk? Does it require a second trigger? Does cyanuric acid accompany adulterated melamine?

Because the toxic dose of melamine is very high (comparable to table salt), the cause of this sudden spurt of cases is undetermined, but the increase in cases is unlikely to result from cumulative melamine burden as the compound is readily cleared by the kidney[6]. Melamine becomes undetectable in rat tissues just 24 hours following exposure to the compound[7].

So, as we await greater scientific understanding of this new man-made disease entity, melamine should be avoided. Melamine is not a bona fide nitrogen-containing nutritional protein, but a mala fide one.

*Rakesh Lattupalli, M.D.*
*Anuradha Kolluru, M.D.*
*Jerry Yee, M.D.*
*Senior Staff Physicians, Division of Nephrology,*
*Henry Ford Hospital, Detroit, MI*

*Corresponding author.
©2008 with author.
Published by TheScientificWorld; www.thescientificworld.com

### REFERENCES

1. Pet Food Recall (Melamine)/Tainted Animal Feed; http://www.fda.gov/oc/opacom/hottopics/petfood.html
2. Thompson, M.E. et al. (2008) Characterization of melamine-containing and calcium oxalate crystals in three dogs with suspected pet food-induced nephrotoxicosis. *Vet. Pathol.* **45(3),** 417–426.
3. Brown, C.A. et al. (2007) Outbreaks of renal failure associated with melamine and cyanuric acid in dogs and cats in 2004 and 2007. *J. Vet. Diagn. Invest.* **19(5),** 525–531.
4. FDA/USDA Joint News Release: Scientists Conclude Very Low Risk to Humans from Food Containing Melamine; http://www.fda.gov/bbs/topics/NEWS/2007/NEW01629.html
5. Puschner, B. et al. (2007) Assessment of melamine and cyanuric acid toxicity in cats. *J. Vet. Diagn. Invest.* **19(6),** 616–624.
6. Baynes, R.E. et al. (2008) Pharmacokinetics of melamine in pigs following intravenous administration. *Food Chem. Toxicol.* **46(3),** 1196–1200.
7. Mast, R.W. et al (1983) Metabolism, disposition and excretion of [14C]melamine in male Fischer 344 rats. *Food Chem. Toxicol.* **21(6),** 807–810.

**This article should be cited as follows:**

Lattupalli, R., Kolluru, A., and Yee, J. (2008) Nephrotoxicity of mala fide melamine: modern era milk scandal. *TheScientificWorldJOURNAL* **8**, 949–950. DOI 10.1100/tsw.2008.134.
